# Supplementary material for: Tailoring a N-Doped Nanoporous Carbon Host for a Stable Lithium Metal Anode
Source: Nanomaterials (Basel). 2023 Nov 23;13(23):3007. doi: 10.3390/nano13233007 (PMC10707975; doi:10.3390/nano13233007)
Supplement: Supplementary file 1 [file nanomaterials-13-03007-s001.zip › nanomaterials-2700232-supplementary.docx]

**Supporting Information**

Tailoring a N-Doped Nanoporous Carbon Host for a Stable Lithium Metal Anode

In-Hwan Lee ^1,†^, Yongsheng Jin ^1,†^, Hyeon-Sik Jang ^2,^* and Dongmok Whang ^1,^*

^1^ Department of Advanced Materials Science and Engineering, Sungkyunkwan University (SKKU), Suwon 16419, Republic of Korea; koggiree24@skku.edu (I.-H.L.); jinzuyeye@skku.edu (Y.J.)

^2^ School of Semiconductor Science & Technology, Jeonbuk National University,
Jeonju 54896, Republic of Korea

* Correspondence: hsjang88@jbnu.ac.kr (H.-S.J.); dwhang@skku.edu (D.W.)

^†^ These authors contributed equally to this work.


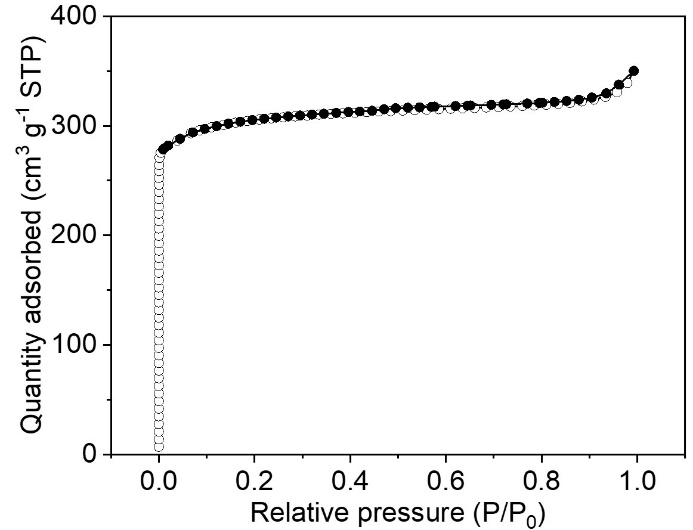


**Figure S1.** N_2_ adsorption-desorption isotherm of CZ-8.


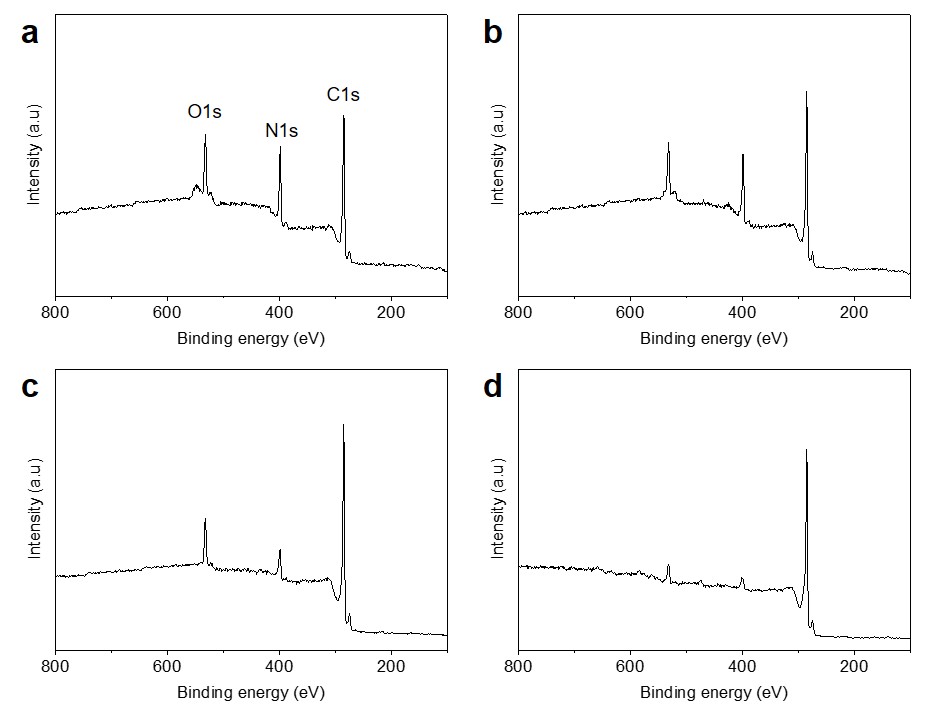


**Figure S2.** The full-scan XPS spectra of (a) CZ-6, (b) CZ-8, (c) CZ-9, and (d) CZ-10.


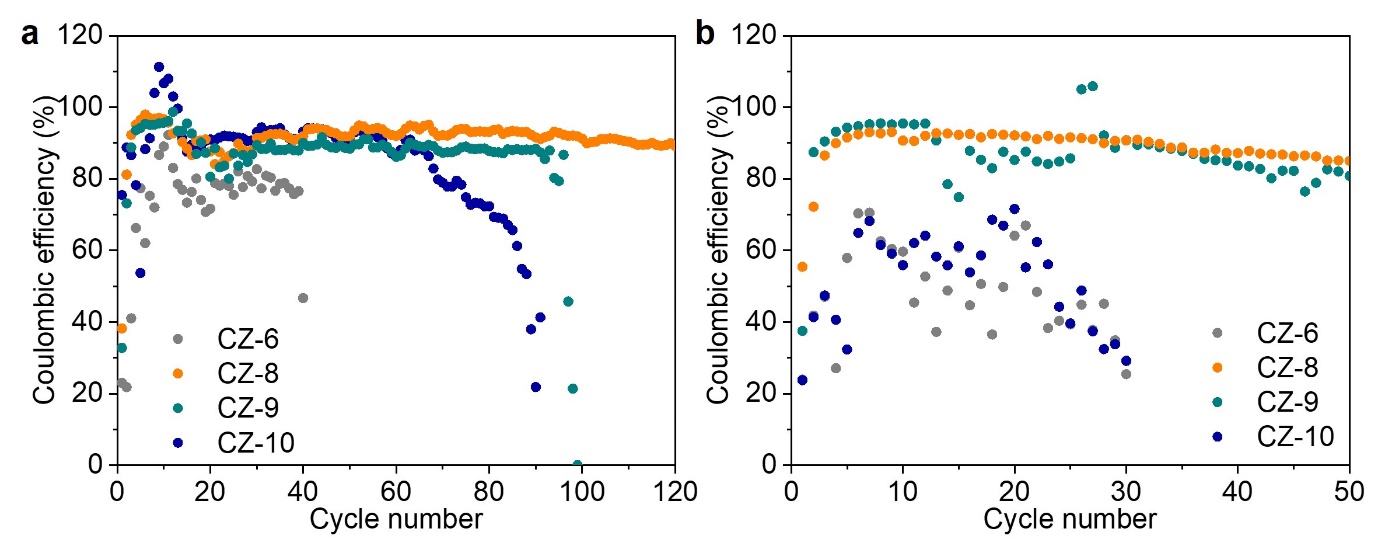


**Figure S3.** Coulombic efficiency of CZ-6, CZ-8, CZ-9, and CZ-10 electrodes at current density of (a) 1 mA cm^-2^, (b) 2 mA cm^-2^.

**Table S1.** Fitting results of all elements of the equivalent circuit model.
